# Nurse-led interventions for self-care and restitutive behaviors in hypertension: A quasi-experimental study

**DOI:** 10.6026/973206300212653

**Published:** 2025-08-31

**Authors:** Anitha Parasuraman, Shankar Shanmugam Rajendran, Gomathy Priya Venkatachalam, Gnana Malar Periyakaruppan, Muthupetchi Ramachandran, Malathi Dhakshnamoorthy, Jebamalar Rajan

**Affiliations:** 1 Department of Community Health Nursing, College of Nursing, Madras Medical College, The Tamil Nadu Dr. MGR Medical University, Chennai, Tamil Nadu, India; 2 Department of Child Health Nursing, College of Nursing, Madras Medical College, The Tamil Nadu Dr. MGR Medical University, Chennai, Tamil Nadu, India; 3 Department of Community Health Nursing, College of Nursing, Madras Medical College, The Tamil Nadu Dr. MGR Medical University, Tamil Nadu, India

**Keywords:** Hypertension, nurse-led intervention, self-care management, restitutive behavior, patient education

## Abstract

The effectiveness of nurse-led interventions on self-care management and restitutive behaviours among hypertensive patients is of interest. Conducted over four weeks in an urban primary health centre in Chennai, 100 participants were divided into experimental and control groups. The experimental group received structured interventions, resulting in significant improvements in behavior and self-care (p < 0.001). The control group showed no significant changes. Thus, we show that nurse-led programs enhance patient engagement and adherence, emphasizing their role in managing hypertension effectively.

## Background:

Hypertension, or elevated blood pressure, is an increasing global health concern and a significant risk factor for cardiovascular illnesses, including myocardial infarction and cerebrovascular accident. Referred to as the "silent killer," it frequently exhibits no symptoms, rendering early detection via routine monitoring crucial [1]. In recent decades, the global prevalence of hypertension has increased significantly. In 1990, around 650 million adults aged 30-79 were affected with hypertension, a figure that nearly doubled to 1.28 billion by 2019, especially in low- and middle-income nations [2]. In the United States, 48.1% of adults are hypertensive or receiving medication, although only 22.5% adequately manage their blood pressure [3]. India exhibits a significant prevalence, with 22.6% of persons affected, rising to 48.4% among individuals aged 60 and older [4]. Alarmingly, over 700 million individuals with hypertension are untreated worldwide, highlighting the urgent want for improved detection and therapy [5]. A major barrier is the deficiency of awareness: approximately 46% of hypertensive individuals are unaware of their disease [6]. Despite being diagnosed, therapy is inadequate; approximately 29.2% of hypertensive persons in the U.S. maintain regulated blood pressure [7]. Medication adherence presents a significant challenge, with 50-80% of patients exhibiting non-adherence attributed to side effects, financial constraints, or intricate regimens [8]. Similarly, lifestyle modifications like adhering to a low-sodium diet or engaging in regular physical activity are challenging to maintain. Effective management of hypertension significantly depends on restitutive behaviors-actions designed to restore and sustain health. This encompasses medication compliance, consistent physical exercise, dietary adjustments and regular blood pressure assessment [9]. Not with standing its significance, compliance with self-care practices is disturbingly low. Only 9.1% of patients exhibit adequate self-care behaviours, and only 19.2% have sufficient information regarding hypertension management [10]. Lifestyle management is essential for controlling hypertension. The world Health Organization advises restricting daily salt consumption to fewer than 5 grammes and enhancing the intake of potassium rich foods, including fruits and vegetables (World Health Organization, 2020) [11]. Engaging in physical activity, namely moderate to vigorous exercise for 150 minutes per week, has been demonstrated to substantially reduce blood pressure. Non-compliance with medicine increases the incidence of cardiovascular events by 50% relative to those who stick to prescriptions [12]. Nurse-led interventions have demonstrated efficacy in enhancing hypertension outcomes. These programs emphasise patient education, lifestyle counselling, and motivational support, thereby markedly improving self-care habits and reducing blood pressure [13]. A systematic study indicated that these therapies decreased systolic blood pressure by 5.39 mmHg and diastolic blood pressure by 1.94 mmHg. Consistent moderate-intensity exercise, such as walking thrice weekly, may lower blood pressure by as much as 7 mmHg [13]. Therefore, it is of interest to describe the impact of nurse-led interventions on improving self-care behaviors and blood pressure management, emphasizing the need for tailored, practical strategies to enhance patient adherence and long-term hypertension control.

## Materials and Methods:

## Study design and population:

A quantitative, quasi-experimental non-randomized control group design was used to evaluate the impact of nurse-led interventions on restitutive behavior and self-care management among clients with hypertension. The study was conducted over four weeks at the NCD Clinic, Choolai Urban Primary Health Centre, Chennai. The target population included all hypertensive clients attending the clinic, while the accessible population comprised individuals who met the inclusion criteria: diagnosed with hypertension, aged ≥35 years, willing to participate, and capable of understanding Tamil or English. Clients with secondary hypertension, recent cardiovascular events, or cognitive impairments were excluded. A non-probability convenience sampling technique was used to recruit participants.

## Sample size calculation:

Based on a prior study by Mekonnen *et al.* (2023) [13], which reported 61.3% adherence to antihypertensive medications, with 95% confidence and 22% relative precision, the required sample size was calculated using the formula: N = (Z^2^ x (1 - p)) / (p x e^2^). This yielded a total of 100 participants, equally divided into experimental (n = 50) and control (n = 50) groups.

## Data collection procedure:

After obtaining ethical approval (No: IEC-MMC/Approval/24112024), and administrative permissions, written informed consent was secured from all participants. A pre-test was conducted using standardized tools: the Health Promoting Lifestyle Profile II (HPLP-II) and Self-Care of High Blood Pressure Inventory (SC-HI V3), along with sociodemographic and clinical questionnaires. The experimental group received structured education and exercise-based interventions, including diet counseling, medication adherence, blood pressure monitoring, diaphragmatic breathing, and 8-walk techniques. The control group received routine care. A post-test was conducted after 21 days using the same tools.

## Data analysis:

Data were coded in Excel and analyzed using SPSS version 26. Descriptive statistics (frequency, percentage, mean, SD) summarized demographics and scores. Chi-square tests assessed demographic similarity and associations. Karl Pearson's correlation tested the relationship between restitutive behaviour and self-care scores. A p-value ≤0.05 was considered statistically significant.

## Results:

The mean age of the participants is 46.07 years, with a standard deviation of 6.31 years. In the experimental group, 26% were aged 46-50 years, 26% were aged 41-45 years, 24% were aged 35-40 years, and 24% were aged 51 years and above. The control group exhibited a comparable pattern, with the most significant percentage (32%) in the 46-50 age groups. Male participants comprised 62% of the experimental group and 50% of the control group. In terms of education, 30% of the experimental group and 34% of the control group had attained higher secondary education, while 22% and 28% respectively, and had completed secondary education. In the experimental group, 44% claimed a monthly income ranging from ₹30,001 to ₹60,000, whereas 42% of the control group reported incomes between ₹10,000 and ₹30,000. The majority of individuals lived in metropolitan regions (94% experimental, 92% control), and nuclear families were the most common family structure (72% experimental, 62% control). At baseline, 88% of experimental participants and 84% of control participants exhibited moderate levels of restitutive behaviour. In the experimental group, 64% exhibited moderate levels of self-care management, while 66% in the control group demonstrated the same. No statistically significant differences were detected between groups at pre-test (p > 0.05). Following the intervention, 68% of experimental participants achieved commendable levels of restitutive behaviour, while 70% acquired satisfactory levels in self-care management. In contrast, the control group exhibited no participants in the favourable category for any area; 26% continued to demonstrate inadequate self-care. The mean restitutive behaviour score in the experimental group increased from 122.78 ± 6.23 to 173.40 ± 5.36, with a mean difference of 50.62 (t = 49.56, p = 0.001). Similarly, self-care management scores improved from 49.20 ± 5.14 to 81.70 ± 6.16 (mean difference = 32.50; t = 24.72, p = 0.001). No significant changes were noted in the control group (Table 1 - see PDF & 2 - see PDF). In the experimental group, age and gender exhibited significant correlations with both outcomes (p < 0.05). Participants aged 51 years and older, as well as females, attained superior scores. The lack of prior surgical history was substantially correlated with enhanced results (p < 0.01). No correlations were identified in the control group.

## Discussion:

This study assessed the effects of nurse-led interventions on restitutive behaviour and self-care management in hypertension patients. Baseline assessments revealed that both the experimental and control groups primarily displayed moderate levels of restitutive behaviour and self-care management, supporting the findings of Jariyasakulwong *et al.* (2025), who identified health literacy and socioeconomic barriers as significant determinants of adherence [14]. Sahile *et al.* (2023) observed inadequate physical activity despite satisfactory medication adherence among Ethiopian patients, underscoring the necessity for comprehensive self-care interventions [15]. Following the intervention, the experimental group demonstrated a considerable enhancement in both outcomes, exhibiting statistically significant improvements relative to the control group. This discovery confirms prior research by Rakhshani *et al.* (2024), which revealed that structured educational interventions grounded in behavioural models markedly improved knowledge, attitudes, and self-care practices [16]. Research conducted by Boima *et al.* (2024) supports the efficacy of digital and home-based self-care education in enhancing hypertension management outcomes [17]. The study indicated that enhancements were attributable not solely to demographic or clinical characteristics, but rather to the specific nurse-led treatments implemented. Age, gender, and surgery history exerted significant influence; however, education and occupation were not reliably predictive. This tendency is corroborated by Hailu *et al.* (2020), who emphasised the complex interaction of information, support networks, and perceptions in shaping self-care behaviours [18]. This study emphasises the essential function of nurse-led interventions in improving patient behaviour, enhancing clinical outcomes, and addressing deficiencies caused by demographic and systemic healthcare constraints.

## Conclusion:

The crucial role of nurses in managing hypertension through education and support is of interest. Nurse-led interventions significantly improved self-care behaviors and blood pressure control. Promoting nurse-led programs can lead to better health outcomes and more effective chronic disease management.
